# Developing an Evidence and Theory Based Multimodal Integrative Intervention for the Management of Renal Cachexia: A Theory of Change

**DOI:** 10.3390/healthcare10122344

**Published:** 2022-11-22

**Authors:** Carolyn Blair, Adrian Slee, Andrew Davenport, Denis Fouque, William Johnston, Kamyar Kalantar-Zadeh, Peter Maxwell, Clare McKeaveney, Robert Mullan, Helen Noble, Sam Porter, David Seres, Joanne Shields, Ian Swaine, Miles Witham, Joanne Reid

**Affiliations:** 1 School of Nursing and Midwifery, Queen’s University Belfast, Belfast BT9 7BL, UK; 2 Division of Medicine, Faculty of Medical Sciences, University College London, London WC1E 6BT, UK; 3 UCL Department of Renal Medicine Royal Free Hospital, University College London, London WC1E 6BT, UK; 4 Division of Nephrology, Dialysis and Nutrition, Hôpital Lyon Sud and University of Lyon, 69495 Lyon, France; 5 Renal Arts Group, School of Nursing and Midwifery, Queen’s University Belfast, Belfast BT9 7BL, UK; 6 Irvine Division of Nephrology, Hypertension and Kidney Transplantation, University of California, Orange, CA 92868, USA; 7 Centre for Public Health, Queen’s University Belfast, Belfast BT12 6BA, UK; 8 Renal Unit, Antrim Area Hospital, Northern Health & Social Care Trust, Antrim BT41 2RL, UK; 9 Department of Social Sciences and Social Work, Bournemouth University, Bournemouth BH12 5BB, UK; 10 Institute of Human Nutrition and Department of Medicine, Columbia University Irving Medical Center, New York, NY 10032, USA; 11 Regional Nephrology Unit, Belfast City Hospital, Belfast Health & Social Care Trust, Belfast BT9 7AB, UK; 12 School of Human Sciences, University of Greenwich, London SE9 2UG, UK; 13 AGE Research Group, NIHR Newcastle Biomedical Research Centre, Newcastle upon Tyne Hospitals NHS Foundation Trust and Newcastle University, Newcastle NE4 6BE, UK

**Keywords:** cachexia, kidney disease, renal disease, end-stage kidney disease, theory of change, personal and public involvement, complex intervention, multimodal intervention

## Abstract

In this study, we aimed to develop a theoretical framework for a multimodal, integrative, exercise, anti-inflammatory and dietary counselling (MMIEAD) intervention for patients with renal cachexia with reference to how this addresses the underlying causal pathways for renal cachexia, the outcomes anticipated, and how these will be evaluated. We used a Theory of Change (ToC) approach to guide six steps. Step 1 included inputs from a workshop to obtain key stakeholder views on the potential development of a multimodal intervention for renal cachexia. Step 2 included the findings of a mixed-methods study with Health Care Practitioners (HCPs) caring for individuals with End Stage Kidney Disease (ESKD) and cachexia. Step 3 included the results from our systematic literature review on multimodal interventions for cachexia management. In step 4, we used the body of our research team’s cachexia research and wider relevant research to gather evidence on the specific components of the multimodal intervention with reference to how this addresses the underlying causal pathways for renal cachexia. In steps 5 and 6 we developed and refined the ToC map in consultation with the core research team and key stakeholders which illustrates how the intervention components of MMIEAD interact to achieve the intended long-term outcomes and anticipated impact. The results of this study provide a theoretical framework for the forthcoming MMIEAD intervention for those with renal cachexia and in subsequent phases will be used to determine whether this intervention is effective. To the best of our knowledge no other multimodal intervention trials for cachexia management have reported a ToC. Therefore, this research may provide a useful framework and contribute to the ongoing development of interventions for cachexia management.

## 1. Background

Cachexia is “a complex metabolic syndrome associated with underlying illness and characterized by muscle loss, with or without loss of fat” [1]. There are various forms of loss of lean muscle mass or wasting in end stage kidney disease (ESKD) including renal cachexia and protein energy wasting (PEW) [2,3]. In ESKD, renal cachexia and the PEW syndrome are closely related, as recent understanding indicates that PEW corresponds to the initial state of a continuous process that progresses to cachexia [4,5,6,7]. Intensive dietary support and dialysis can reverse the deterioration in nutritional status for individuals with ESKD who have anorexia-induced insufficient energy intake [6,8,9,10]. However, improving the nutritional status and body composition in individuals who have renal cachexia is much more difficult because of additional, profound metabolic alterations [6,8,9,10]. In ESKD, up to 75% of adults undergoing maintenance dialysis present with some evidence of wasting [11]. The effects of cachexia are profound, including reduced physical function and increased symptom burden affecting quality of life and increased mortality. Despite the prevalence and consequences of cachexia, currently, no licensed treatment exists [3,12,13,14]. Although there is limited research relating to the medical burden and cost of this debilitating syndrome, in a study using data from one year (2009) of the Nationwide Inpatient Sample (NIS) Arthur et al. [15], suggests that patients with cachexia had twice the number of inpatient days and double the hospitalisation costs when compared with patients without cachexia. It is evident that cachexia is a major contributor to morbidity and to healthcare costs therefore there is urgency to trial interventions which have real possibility of counterbalancing these costs while also improving outcomes for this vulnerable population. Patients with or at risk of renal cachexia actively need interventions which help to increase their capacity for physical functioning, build lean muscle mass, maintain or increase their weight and stabilise abnormal biochemistry to increase their quality of life and prolong survival [2,3,12,13,14,16,17].

In ESKD findings relating to multimodal trials are limited and evidence suggests that isolated interventions in the form of protein supplementation and nutritional energy have rarely proven to be very effective in improving outcomes [18]. The lack of improvement is likely to be due to the need to effectively treat other causative factors (e.g., inflammation or inflammatory burden) [19,20]. For optimal treatment of renal cachexia evidence suggests that a multimodal approach which includes exercise [21,22], anti-inflammatory agents [9,11] and nutritional support [1,8,11] is essential to target the multifactorial pathophysiology and attain at least additional, if not synergistic effects [18]. Multimodal interventions including exercise, dietary counselling and anti-inflammatory agents have proved feasible with beneficial effects in cancer cachexia [23,24,25]. Similarly, the evidence base is clear for cancer cachexia that unimodal interventions have mixed results which warrants the need for a more comprehensive approach to managing cachexia and the development of clinical guidelines for a licensed treatment [25,26,27]. Despite recent evidence demonstrating positive outcomes, multimodal interventions for cachexia management are complex interventions [28] and it is unclear what the most effective elements of the intervention are and in what circumstances it can best be implemented for those with renal cachexia. While there is literature outlining the impact of multimodal interventions and one study, the MENAC (Multimodal—Exercise, Nutrition and Anti-inflammatory medication for Cachexia) trial [25], reporting a rationale for their multimodal intervention relating to cancer cachexia, to date, none have provided a Theory of Change (ToC) to determine the underlying causal pathways for cachexia and the outcomes anticipated through their intervention. 

Frameworks such as those from the Medical Research Council (MRC) on developing and evaluating complex interventions, the Template for Intervention Description and Replication (TIDieR) checklist for better reporting of interventions and the MORECare statement articulate the importance of ‘theory’ and advise that it is best practice to report on the ToC prior to piloting and evaluation to ensure the context and the effective elements of the intervention are understood [28,29,30,31,32]. A ToC is distinctive from other approaches as it not only focuses on how to influence social change by empowering individuals, such as Personal and Public Involvement and Engagement (PPIE) representatives, but it also explains the hypothetical causal pathways through which change may happen [33,34]. Based on Funnell et al., [35] the MRC’s [32] guidance advises that when developing a theory various factors must be taken into consideration including the key components and mechanisms of the intervention and how they interact, the features of the context that are anticipated to influence those mechanisms, and how those mechanisms might influence the context. 

This paper presents the development of a ToC for a proposed multimodal, integrative, exercise, anti-inflammatory, dietary counselling and psychosocial intervention for the management of cachexia in those with ESKD. Through providing an in-depth understanding of how this multimodal intervention is theorised to work and which components are the most important in achieving impact we aim to reach a stronger external validity [36,37]. As advised by the new MRC guidelines [32] we have developed the intervention theory collaboratively with involvement from diverse stakeholders, it is based on evidence and theory from relevant fields, and plan to refine it during successive phases. 

## 2. Materials and Methods

### 2.1. Aim

In this study, we aimed to develop a theoretical framework for a multimodal, integrative, exercise, anti-inflammatory and dietary counselling (MMIEAD) intervention for those with renal cachexia with reference to how it addresses the underlying causal pathways for renal cachexia, the outcomes anticipated, and how these will be evaluated. This serves as a critical step in the development of a multimodal intervention which promotes positive outcomes for those with renal cachexia. 

### 2.2. Design

**Figure 1:**
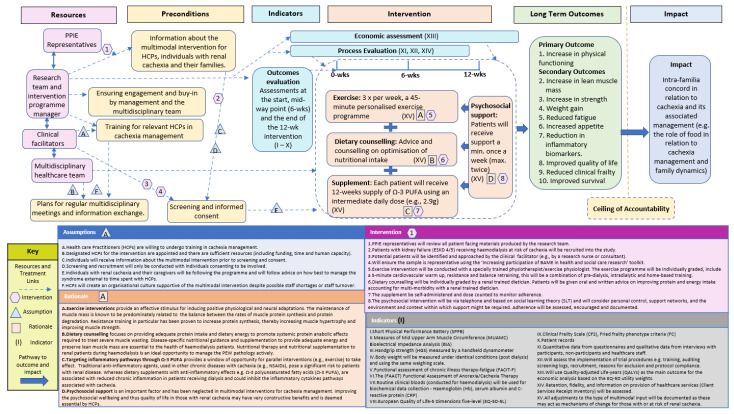
ToC Map.

A ToC approach was used to develop a multimodal intervention for cachexia management. Following the Aspen Institute and Centre for Theory of Change, a ToC is “a theory of how and why an initiative works which can be empirically tested by measuring indicators for every expected step on the hypothesised causal pathway to impact” [33,38]. We used input from a range of key multidisciplinary stakeholders including an international collaboration of consultants and academics specialising in nephrology, nutrition, exercise, psychology and PPIE representatives. To further build the evidence base for our ToC Map we conducted a mixed-methods study (online survey and two focus groups) with Health Care Practitioners (HCPs) with ESKD and cachexia [12] and a systematic literature review on multimodal interventions [13]. Our wider cachexia research which includes cancer cachexia [39,40,41,42,43,44], cardiac cachexia [16,45,46,47,48] and renal cachexia [2,3,12,17] was also used alongside relevant renal literature to build evidence to construct a multimodal intervention for renal cachexia. The ToC approach is visualised in a ToC map (Figure 1), which provides a comprehensive illustration of how long-term outcomes can be achieved through the MMIEAD intervention [33]. Within this map specific terms are used (Table 1).

In this paper, we illustrate the process of developing a ToC map as part of the development phase of the multimodal intervention. De Silva et al. [33] suggest that this process has the potential to strengthen the MRC framework in its key stages of development, feasibility/piloting, evaluation and implementation. Given that we wanted to enhance stakeholder engagement, improve the initial design of the intervention, and identify any potential barriers to implementation a ToC approach was deemed to be fitting in the development phase with the prospect of revising after the feasibility/piloting, evaluation and implementation phases [28]. To create our ToC map we used “backwards outcome mapping”, starting by drafting the impact and long-term outcomes that are to be achieved then, working backwards to define the long term outcomes, intervention, indicators, preconditions and resources [37].

## 3. Steps to Develop the ToC Map

We undertook six steps to develop the ToC map (Table 2). Step 1 included inputs from a workshop to obtain key stakeholder views on the potential development of a multimodal intervention for renal cachexia. Step 2 included the findings of a mixed-methods study with HCPs caring for ESKD patients and those with cachexia. Step 3 included the results from our systematic literature review on multimodal interventions for cachexia management. In step 4, we used the body of our research team’s cachexia research and wider relevant research to identify the specific components and hypothesised causal pathways for renal cachexia. In steps 5 and 6 we developed and refined the ToC map which illustrates how the intervention components of MMIEAD interact to achieve the intended impact of improved functionality and quality of life and reduced premature mortality.

### 3.1. Step 1: Obtain Key Stakeholder Views on the Potential Development of A Multimodal Intervention for Renal Cachexia 

The workshop included a range of key multidisciplinary stakeholders including an international collaboration of consultants and academics specialising in nephrology, nutrition, exercise, psychology and PPIE representatives. The PPIE representatives provided expert patient knowledge and experience that reinforced the acceptability of a multimodal intervention however expressed the need for due diligence in relation to concern about patient burden. Consultants also confirmed acceptance of the need for a multimodal intervention given their clinical experience, however emphasised the need to fully investigate the evidence which suggests that this multimodal intervention has the potential to be more effective than current treatments. All stakeholders at the workshop agreed that they hoped the multimodal intervention would achieve would improve functionality, quality of life and reduce premature mortality. The distress caused in families through difficulties in cachexia management was also raised as a concern which they hoped a multimodal intervention would impact. Emphasis included the necessity that care planning is discussed amongst multidisciplinary HCPs to minimise patient burden and ensure that multidisciplinary care is a seamless as realistically possible. Consultants also indicated that to be involved in recruiting patients for a trial such as this they would need full support from the research team. The importance of information sessions with individuals with renal cachexia and their caregivers was also highlighted as a priority in order for individuals and their families to understand the condition as fully as possible and why a multimodal intervention is needed. The workshop was initially conducted to ascertain the ‘buy-in’ of representatives who are essential to guide the development of a multimodal intervention. Collaboration with this group of key stakeholders also provided essential information to inform the impact, preconditions and the hypothesised causal pathways for those with renal cachexia. Furthermore, our PPIE representatives and other stakeholders also helped to identify key outcome measurements that reflected patient needs (e.g., increased quality of life, reduced fatigue, increase in functionality and strength). During this workshop, a lead PPIE representative, from Kidney Care UK and Northern Ireland Kidney Patients Association (NIKPA) agreed to advocate the need for progressing the work and stay in close contact with the research team during the development of the intervention. The stakeholders subsequently agreed to work collaboratively with the core research team to provide further feedback on the development of the proposed intervention, ToC map and selected outcome measures.

### 3.2. Step 2: Obtain HCP Perspectives on Current Practices in Cachexia Management

In our mixed method study on awareness, understanding and treatment practices when managing cachexia in ESKD [12], HCPs reported the management of cachexia as complex and challenging compounded by a lack of guidelines or standards of practice for the treatment of renal cachexia. This evidence further supports the need for a disease specific intervention for renal cachexia reinforced by the fact that models of care for cancer patients with cachexia were regarded by HCPs as good examples that could be adapted for the management of patients with ESKD [12]. HCPs identified multiple salient issues that are particularly relevant to the development of an intervention for those with renal cachexia, i.e., related behavioural factors which may influence decision-making for example in relation to nutrition or exercise choices. In particular, HCPs emphasised their concern regarding the distress experienced by patients with renal cachexia and caregivers alike [12]. It was acknowledged by HCPs that “information material [relating to renal cachexia] is scarce or absent” and this lack of information can mean that individuals with renal cachexia are “very frightened” about changes in appetite that may be correlated with cachexia [12]. Addressing the underlying factors through psychosocial support was identified as a necessity to decrease distress and improve quality of life [12]. In response we have incorporated social learning theory (SLT) [70] to identify how change could be influenced to good effect in building this proposed evidence-based intervention. SLT is pertinent to health behaviours in those with renal cachexia considering that personal control, support networks, the environment and context within which support might be required are all vitally important [12,70]. We anticipate that through incorporating SLT we may be able directly influence adherence to the intervention which would inevitably have very constructive benefits, for example, having the motivation and confidence to participate in exercise programmes combined with the capacity for self-care to plan protein rich meals may have a significant effect on health status. Theories on change mechanisms acknowledge that underpinning and linking interventions to a relevant theory can help to identify how and why change can be influenced [33]. In order to increase the psychosocial wellbeing and thus quality of life in those with renal cachexia HCPs also recognised a need to more fully understand the multifactorial nature of cachexia and apply it consistently in clinical practice [12]. HCPs also deemed patient-clinician communication to help dispel confusion around renal diet restrictions coupled with cachexia as crucial [12]. Through this mixed method study, we identified that given the lack of disease specific guidelines increased awareness and training for HCPs is an essential pre-requisite and precondition in the development of the ToC Map. Furthermore, a psychosocial component to the intervention is critically important to decrease intra-familia distress, encourage and support willing adherence to the intervention. 

### 3.3. Step 3: Identify and Assess the Effectiveness of Multimodal Interventions for Cachexia Management

Our systematic review in step 3 was based on examining multimodal treatments intended to alleviate and/or stabilize cachexia and severe wasting across chronic diseases [13]. We found that all included studies on chronic obstructive pulmonary disease (COPD) [54,55,56,57,71], ESKD [22,72,73,74] and cancer [23,49,50,51,52] demonstrated some improvement in favour of the treatment groups. In cancer, research in multimodal interventions has progressed further than other chronic diseases, which provides evidence relating to the need to further research in ESKD and the potential usefulness of this approach for cachexia management [13,23,49,50,51,52,53]. The synthesis for our review was based on Evans et al.’s [1] definition of cachexia (weight, functional status, nutritional status, biochemistry). In order to determine how our review findings could further enhance the development of an effective, relevant, and sustainable intervention, we also focused attention on the patient experience (e.g., mortality, tolerability, quality of life) [13]. Our review confirms that there are greater improved endpoints when combining treatment modalities, which furthers our understanding of and confidence in the effectiveness of multimodal interventions for cachexia management [13]. Given that the majority of studies found significant improvements in weight gain, body composition, and physical activity as well as functionality [50,51,54,55,56,57] this influenced our choice of long-term outcomes and subsequent choice of measures. Notably, however our review highlighted that no studies reported psychosocial interventions; however, several studies stated Quality of life (QoL) endpoints [13]. The review also highlighted that randomised controlled trials (RCTs) with a powered sample size and sufficiently lengthy interaction period are necessary to assess if multimodal interventions are effective forms of therapy for improving body composition and nutritional and physical status in patients with cachexia and wasting [13]. Overall, the evidence from this review supports the role of multimodal interventions in the treatment of cachexia. In consultation with key stakeholders, we used the studies which showed promise regarding their study design and results [50,51,54,55,56,57] to inform our intervention design and proposed long-term outcomes.

### 3.4. Step 4: Develop and Refine the Components of the Proposed Multimodal Intervention for the ToC Map

In consultation with key stakeholders, we used the existing evidence from our body of research team’s cachexia research (cancer cachexia [39,40,41,42,43,44], cardiac cachexia [16,45,46,47,48] and renal cachexia [2,3,12,17]) and wider evidence to assist in providing context and a rationale for the components of the multimodal intervention for renal cachexia.

i. Exercise interventions—Loss of skeletal muscle mass can cause loss of independence, disability, and an increased risk of morbidity and mortality [58,59,60,75]. Hence why, the maintenance of muscle mass has been recognised as a determinant which directly influences quality of life [76]. There is a strong rationale for the use of exercise interventions in regulating both catabolic and anabolic pathways and modulating levels of inflammation in cancer cachexia [77,78]. In ESKD, is more limited however evidence to date suggest that resistance exercise might be more effective in specifically combating loss of physical function and functional decline including the loss of independence in activities of daily living [59,60]. Frailty intervention studies [79,80] for those on haemodialysis also show that improved functioning can be improved with exercise which thereby contributes to reducing frailty. The maintenance of muscle mass is known to be predominately related to the balance between the rates of muscle protein synthesis and protein degradation [76,81]. Evidence suggests that the recovery of muscle mass and function is much greater from resistance exercise, than aerobic exercise and it increases protein synthesis and occurs more rapidly for this type of exercise [58,82,83,84,85,86,87]. Within the renal population, studies suggests that 45 min of moderate intensity [88] exercise 3 times per week is safe and tolerable [89,90]. Despite some positive evidence supporting the case for exercise, it is necessary to state that given the quality of the studies to date, a recent Cochrane review [91] on cancer cachexia and exercise found insufficient evidence to support or refute the use of exercise alone, or as part of a complex multimodal intervention. However, Grande et al. [91] in accord with Ferran et al. [92] state that rigorous and better designed studies with a clear theoretical reasoning in this area are deemed essential to help patients and clinicians decide if exercise could be beneficial [91].

ii. Dietary counselling—Chronic disease-related malnutrition in ESKD is a complex issue given that disease specific factors such as inflammation must also be taken into consideration [19,20]. Koppe, Fouque & Kalantar-Zadeh [6] confirm that PEW is among the strongest predictors of mortality in patients with ESKD (hazard ratio of 3.03; confidence interval of 1.69–5.26 in 1068 haemodialysis patients). Dietary counselling focuses on providing adequate protein intake and dietary energy to promote systemic protein anabolic effects required to treat severe muscle wasting [93]. Evidence suggests that providing nutrition counselling could be linked to improvements in the patients’ malnutrition inflammation scores and nutritional knowledge and status [61,62]. Disease-specific nutritional guidance and supplementation to provide adequate protein, energy and preserve lean muscle mass is therefore essential to the health of haemodialysis patients [19,94,95,96,97,98,99]. Evidence suggests that nutritional therapy and nutritional supplementation to renal patients during haemodialysis is an ideal opportunity to manage the PEW pathology actively and help to manage one of the most critical risks for mortality in haemodialysis patients [94,95,96,97,98,99]. 

iii. Targeting inflammatory pathways through Ω-3 PUFA—Cachexia development is highly dependent on the immune response, whereby proinflammatory cytokines are largely responsible for enhanced muscle degradation and loss of appetite, even in the face of adequate nutrition [100]. Ω-3 PUFAs are suggested to be promising and well-studied pharmaconutrients in cancer cachexia and have improved appetite and body weight when incorporated into high protein and energy oral nutritional supplements (ONS) [101,102,103,104]. Across other chronic diseases, there is limited research, however Calder et al. [54] trialled Ω-3 PUFAs in combination with an ONS in patients with COPD and reported several positive effects including weight gain, improved body composition (e.g., fat mass), functionality (e.g., fatigue, dyspnoea), and metabolic biomarkers (e.g., blood pressure, lipoprotein, and cholesterol). There is general consensus that multimodal approaches to the treatment of cachexia should include anti-inflammatory agents [100,105] and ongoing research investigating how cytokines such as IL-6, TNF-α and IL-1β that trigger inflammatory cascades may play a role in the pathogenesis of renal cachexia [106]. However, there has been debate whether inflammatory status affects the response to nutritional support, in one of the largest nutritional intervention studies in maintenance haemodialysis (MHD) patients with PEW (FINE study), 186 MHD patients with PEW were enrolled and received either intradialytic parenteral nutrition (IDPN) plus ONS or ONS alone [8]. Similar improvements of nutritional parameters were observed in the two groups over two years and there were no differences in rates of hospitalisation or death [8]. The clinically relevant conclusions were that the inflammatory status does not significantly affect the response to nutritional support [8]. Traditional anti-inflammatory agents, used in other chronic diseases with cachexia (e.g., Non-steroidal anti-inflammatory drugs (NSAIDs) [23]), pose a significant risk to patients with renal disease [107]. Whereas dietary supplements with anti-inflammatory effects, e.g., Ω-3 polyunsaturated fatty acids (Ω-3 PUFA), are associated with reduced chronic inflammation in patients receiving dialysis and could inhibit the inflammatory cytokines pathways associated with cachexia [108,109]. The rationale for Ω-3 PUFA is based on multiple studies showing cardioprotective effects and the effectiveness on inflammatory markers, lipid profile and heart rate [63,64,65,66,67]. A recent meta-analysis also adds knowledge to the existing literature suggesting that Ω-3 PUFA supplementation (in the magnitude of around 3 g per day) may reduce the risk of cardiovascular death in patients treated with haemodialysis [110]. Aside from minimal gastrointestinal side effects (e.g., fishy aftertaste), evidence suggests that Ω-3 PUFA consumption at the suggested dose do not cause other serious adverse effects [110]. Although there is limited evidence of the effectiveness of using Ω-3 PUFAs in a multimodal intervention for renal cachexia, given the safety profile in the renal population, and effectiveness in other chronic conditions this is the most promising option to help increase appetite, body weight and reduce inflammation.

iv. Psychosocial support—Psychosocial issues in individuals with ESKD patients are frequently overlooked yet are often modifiable risk factors for mortality [68]. It is important to acknowledge that when a patient’s relationship with food is negatively affected, this impacts social and family aspects. Therefore, the proposed intervention should address the emotional and social context of such factors likely to impact eating problems, such as distress, anxiety, and support for family carers [41]. Providing psychosocial support is a critically important consideration in designing this multimodal intervention [23]. This is something that needs to be understood, both by individuals at risk and those with renal cachexia and those who care for them. As noted, in this intervention, it is important to consider SLT, as there are several salient issues that are especially relevant to those with chronic diseases, i.e., related behavioural factors may influence decision-making in specific respects during this time (e.g., nutritional and functional status). For example, having the motivation and confidence to participate in exercise programmes combined with the capacity for self-care to plan protein-rich meals may have a significant effect on health status. Although evidence is limited, social support has also been deemed to be an important aspect of in reducing fatigue experience for individuals with ESKD, serving as a source of motivation [69]. As advised within the recent Cochrane review on exercise and cancer cachexia, health behaviour changes strategies should be considered as these may be required to optimise adherence [91]. Evidence suggests that those who are supported in and advised to self-monitor exercise and nutritional intake are more successful at changing behaviours which lead to positive outcomes regarding than those who are inconsistent with self-monitoring [21,111]. Considering the evidence available, psychosocial support may have very constructive benefits in improving the psychosocial wellbeing and thus quality of life in those with renal cachexia [12,68,69].

Integrating all these factors into an intervention for those with renal cachexia is novel and will help to ensure that influential elements and contexts are taken into account and increase the possibility that the components of the programme function synergistically. For example, targeting inflammatory pathways provides a window of opportunity for parallel interventions (e.g., exercise) to take effect. Using the synthesised evidence from Step 1–4, in collaboration with key stakeholders, we drafted the intervention design for the ToC map and the rationales, to illustrate how an intervention might be designed to impact positively upon stabilising or reversing the impact of renal cachexia.

### 3.5. Step 5: Create a Draft ToC Map Based on Integration of Output from Steps 1–4

To refine the proposed intervention and ensure that the intervention has considered all factors which may influence intervention delivery the core research team designed a ToC map. In designing the ToC map, we have used the synthesised evidence from Steps 1–4 to identify how a multimodal intervention for cachexia management could be designed to be most effective. To ensure that the findings of its delivery are evidence based and replicable in other contexts the steps for the outcomes, process and economic evaluation were added into the ToC Map. In the ToC Map we include reference to the assumptions, rationale, preconditions, indicators, components and duration of such input. We also have indicated where there may be variation in intervention delivery and the necessity of recording this information for example, taking into account the effectiveness of cardiovascular activity orientated exercise programmes compared to resistance programmes. It may be that adjustments to the type of multimodal input may act as mechanisms of change for those with or at risk of renal cachexia. Based on Steps 1–4, the core research team drafted the full ToC map: (i) impact, (ii) ceiling of accountability, (iii) long-term outcomes, (iv) preconditions, (v) interventions, (vi) assumptions, (vi) rationale (vii) indicators and (viii) resources. In this map we illustrated the proposed full-scale intervention and evaluation plan building on the previous steps and aimed to incorporate, rather than duplicate information. The ToC Map and associated documentation was then disseminated to the key stakeholders with a request for feedback given their various areas of expertise.

### 3.6. Step 6: Develop and Amend the ToC Map in Consultation with Key Stakeholders

Post in-depth consultation with key stakeholders and the core research team, further integration of the outputs from steps 1–5 and further guidance have been incorporated into a further draft of a ToC map. This included guidance to:

- add an emphasis on the integrative nature of the intervention;
- clarify the hypothesised causal pathway for those with renal cachexia and the proposed impact of the multimodal intervention;
- clarify the rationale for the components of the multimodal intervention;
- highlight the importance of the psychosocial component and how this will be integrated into the intervention in practice;
- merge, reformulate and put the preconditions in chronological order;
- clarify the proposed outcomes and add clinical frailty as a secondary outcome;
- simplify the rationale of the exercise component to more user-friendly language;
- eliminate repetition where possible;
- ensure sufficient understanding of the importance of equipoise when planning the MMIEAD intervention;
- revise the proposed impact based on the evidence to date and the ceiling of accountability.

Post amendments, the ToC Map and associated documentation was circulated to key stakeholders again to ensure all comments and critique had been suitably amended/responded to. The final ToC map (Figure 1) and associated explanation is described as follows. 

## 4. Results

We followed the Checklist for reporting ToC in Public Health Interventions [112] (Supplementary Table S1) and have documented the following in diagrammatic form (Figure 1): (i) impact, (ii) ceiling of accountability, (iii) long-term outcomes, (iv) preconditions, (v) interventions, (vi) assumptions, (vi) rationale (vii) indicators and (viii) resources. The following Toc Map and explanatory material will focus predominantly on the intervention for those with renal cachexia with reference to how it addresses the underlying causal pathways for renal cachexia, the outcomes anticipated, and how these will be evaluated. An intervention flow chart for a cluster randomised controlled trial (cRCT) using the 12-week multimodal intervention with an internal pilot, process evaluation and economic evaluation in six NHS renal units is illustrated and explained in Supplementary Document 2. 

### 4.1. Impact

The desired impact that should be achieved for those with renal cachexia was identified as ‘Intra-familia concord in relation to cachexia and its associated management (e.g., the role of food in relation to cachexia management and family dynamics)’. Through steps 1–4 and in particular the mixed methods HCP study [12] we have evidence to suggest that that reducing family distress in relation to cachexia management is a vitally important part of cachexia management and therefore the desired impact of this proposed intervention.

### 4.2. Ceiling of Accountability

The threshold at which the multimodal intervention is no longer directly accountable for the desired impact is delineated by the ‘ceiling of accountability’, which is situated between the impact stated above and the long-term outcomes listed below. Although we aspire to influence intra-familia concord in relation to cachexia and its associated management (e.g., the role of food in relation to cachexia management and family dynamics) it is not possible to measure the desired impact using the proposed research approach and therefore beyond the accountability of this project. Given that this protocol has been designed as a patient intervention not a family intervention we do not plan to measure whether this intervention will reduce distress in families. Although the multimodal intervention is very likely to contribute to achieving the desired impact through its effect on the long-term outcomes, we acknowledge that the point, illustrated in Figure 1, is the ceiling of accountability.

### 4.3. Long-Term Outcomes and Indicators

Based on Evans et al.’s [1] definition of cachexia, the results of the stakeholders’ views, the contextual analysis, systematic review and defining the intervention [2,3,12,13,17] we identified ten long-term outcomes that are desired to be achieved by the multimodal intervention. The outcomes have been merged with the indicators in Table 3: Long-term outcomes and measures to show directly how these outcomes will be measured.

Other indicators will include (explained more fully in Supplementary Document 2: Intervention flow chart):

The process evaluation will include analyses of quantitative data from questionnaires and qualitative data from interviews with participants, non-participants and HCPs. 

The implementation of trial procedures (e.g., training, auditing screening logs, recruitment, reasons for exclusion and protocol adherence) will be logged and assessed.

Quality-adjusted Life-years (QALYs) will be used as the main outcome for the economic analysis based on the EQ-5D utility weights.

Retention, fidelity, and information on provision of healthcare services (Client Services Receipt Inventory) will be assessed.

All adjustments to the type of multimodal input will be documented as these may act as mechanisms of change for those with or at risk of renal cachexia.

### 4.4. Rationales

In Figure 1, we describe (in summary form) the rationales for the components of the intervention, in particular drawing from the evidence in Steps 3 and 4. Although specific components of the intervention are proposed to have an influence on specific long-term outcomes, we emphasise that for the intervention to work successfully it is by necessity that these component parts are integrative. 

### 4.5. Interventions

The multimodal intervention will be designed as follows: In addition to their usual haemodialysis standard care, participants will receive an individualised graded resistance exercise and dietetic programme combined with oral Ω-3 PUFA supplementation. Patients will be asked to keep exercise and food diaries to monitor adherence to initiated changes and will be followed up prospectively (using face-to-face appointments and telephone reviews) to support and monitor progress. 

i. Exercise: Each patient will receive 3 × per week, a 45 min personalised exercise programme over a 12-week period with a specially trained physiotherapist/exercise physiologist. The exercise programme will include a combination of pre-dialysis, intradialytic and home-based training. The exercise intervention will consist of a 45 min programme moderate-intensity exercise at least three times per week. This will include basic aerobic cardiovascular exercise (i.e., 5–10 min warm up), muscle strengthening through resistance training and balance retraining exercise. The regime will progress in difficulty and each participant will receive an instruction booklet and diary to complete. 

ii. Dietary counselling: Nutritional counselling with advice on optimization of nutritional intake will be provided by a renal trained dietician. A nutritional interview (30 min) will be performed at baseline, and then patients will be given oral and written advice on improving protein and energy intake accounting for multi-morbidity with a renal trained dietician. 

iii. Supplement: Each patient will receive 12-weeks supply of Ω-3 PUFA using an intermediate daily dose (e.g., 2.9 g; [120]), this will be in oral capsule formulation, self-administered and dose counted to monitor adherence. 

iv. Psychosocial support: Patients will be given psychosocial support via telephone (to ensure sustainability) a minimum of once a week (maximum of twice) pending on adherence to the intervention. In order to decrease burden on the clinical team, the psycho-social support will be provided by a researcher who has a background in psychology and mental health training. The psychosocial support will be based on SLT and will therefore take into account an individual’s self-efficacy (e.g., in terms of confidence, belief, ability, motivation, etc.) [70]. During these sessions adherence to the intervention will be assessed (written notes will be recorded) and engagement with the multimodal intervention will be encouraged.

### 4.6. Preconditions

Based on steps 1–4, we identified five important preconditions that need to be fulfilled for the desired long-term outcomes to be achieved. 

#### 4.6.1. Ensuring Engagement and Buy-in by Management and the Multidisciplinary Team

With multiple factors in favour of the MMIEAD intervention potentially improving outcomes for those with ESKD and a limited evidence base on using single modalities to improve outcomes, there is clinical equipoise to perform a cRCT to compare different strategies to manage renal cachexia. To make sure the clinical leads sufficiently understand the evidence base around the multimodal intervention, are willing to implement the multimodal intervention, the intervention project manager should have regular and sustained meetings with them to explain the study, establish their trust, co-operation, engagement and ensure ‘buy-in’ to the study. Working closely with the clinical team is an essential pre-requisite of this study to optimise recruitment and ensure ethical adherence. Research has shown that establishing management support ensures that if all relevant staff have an understanding of the importance of the intervention and how to support the intervention effectively and appropriately the result that it is more likely to be sustained [121].

#### 4.6.2. Training for HCPs

Training in cachexia management is required to ensure that relevant HCPs are confident and knowledgeable in conducting follow up conversations relating to the multimodal intervention with individuals with renal cachexia and their caregivers. Those with renal cachexia usually have complex health trajectories and this can cause additional anxiety for patients and caregivers, and evidence confirms that conversations relating to cachexia are notably difficult for HCPs [12]. Therefore, designated HCPs for the proposed intervention should have training to enhance their understanding and skills in cachexia management to help them confidently interact with patients and caregivers regarding the multimodal intervention.

#### 4.6.3. Multidisciplinary Meetings and Information Exchange

To ensure that the care planning of all participants is known to all relevant HCPs the multimodal intervention should be regularly discussed in multidisciplinary meetings. The conversations held with individuals with renal cachexia or their caregivers relating to intervention should also be included in the multidisciplinary meetings. Information exchange is anticipated to reduce patient burden and improve HCP teamwork. The importance of teamwork to achieve goals is supported by theories related to team effectiveness [121] as well as the practical experience of the stakeholders and authors.

#### 4.6.4. Information about the Multimodal Intervention for Individuals with Renal Cachexia and Their Families

All HCPs, management, individuals with renal cachexia and their caregivers should be informed about the multimodal intervention using letters and information sessions. Being fully informed about what the multimodal intervention is will help individuals to accept why it is needed, be adequately prepared, make effective decisions as to whether to be involved and/or remain engaged in the intervention. It is also extremely important for those with renal cachexia and their caregivers to be fully informed throughout the process in order to encourage self-management to improve the outcomes of the programme. Potential patients should be identified and approached at the NHS sites by the clinical facilitators (e.g., by a research nurse or consultant). The clinical facilitator should ensure that sample is representative of patients from the Black Asian and Minority Ethnic (BAME) community by using the ‘Increasing participation of BAME in health and social care research’ toolkit [122]. All potential participants approached should receive a study information pack (SIP) containing a patient information leaflet, an invitation letter and a reply slip. The SIP should also include a telephone number and e-mail address in the eventuality that any potential participant would like to discuss the study in more detail with a member of the local research team, before deciding to participate. 

#### 4.6.5. Screening and Informed Consent

The eligibility criteria of participants will be based on Evan’s et al.’s [1] definition of cachexia and expanded upon in the protocol for the pilot and cRCT. Screening and informed consent’ are pre-requisites to involvement in the study, it is essential that eligible participants understand the research and voluntarily agree to take part. A member of the local research team will outline the study, answer any queries, confirm eligibility, and obtain informed consent. 

### 4.7. Assumptions

Assumptions are defined as the contextual conditions that need to be in place for the multimodal intervention to function successfully. A failure to provide these will create barriers which is likely to hinder the achievement of the long-term outcomes. Based on steps 1–6, the assumptions are that: 

HCPs are willing to undergo training in cachexia management and designated HCPs for the intervention are to be appointed. 

There are sufficient resources (including funding, time and human capacity).

Potential participants will receive information about the multimodal intervention prior to screening and recruitment. 

Screening and consent procedures will only be conducted with individuals consenting to be involved. 

Individuals with renal cachexia will adhere to the programme and will follow advice on how best to manage the syndrome external to time spent with HCPs. 

HCPs will create an organisational culture supportive of the multimodal intervention despite possible staff shortages or staff turnover.

## 5. Discussion

Using the Theory of Change approach, we have developed a theoretical framework for a multimodal, integrative, exercise, anti-inflammatory and dietary counselling (MMIEAD) intervention. The ToC map makes explicit the rationale and what long-term outcomes are expected as a result of the intervention and how these will be measured for patients with renal cachexia. MMIEAD is a complex intervention, and the hypothesised causal pathways are not linear, therefore this work emphasises that in order for the long-term outcomes to be achieved all intervention components of MMIEAD must be integrative. Given the evidence gathered through this work, our aspiration is that the MMIEAD intervention will also achieve the intended impact of ‘intra-familia concord in relation to cachexia and its associated management’. As De Silva et al. [37] state ToC can be used to strengthen RCTs by building and validating program theories of interventions that are then empirically tested [123]; therefore the ToC map will subsequently be revised to produce a ‘story’ of how MMIEAD worked during feasibility and pilot testing [33]. 

The approach used in this study has led us to the development of a multimodal intervention which is distinctly different yet shares some key characteristics with those that have been developed before and found significant improvements in weight gain, body composition, and physical activity as well as functionality [50,51,54,55,56,57]. Additionally, important elements were added for a renal population compared to existing multimodal interventions including a psychosocial intervention given the evidence gathered from steps 1–4. Secondly, our ToC map highlights that a change in desired outcomes through a multimodal intervention is hypothesised to be achieved only by targeting multiple factors in an integrative manner. Thirdly, our research confirms that improved collaboration and information exchange with those involved in multidisciplinary care delivery may lead to better outcomes and more effective self-management capacities of patients and informal carers. Hence, when MMIEAD is delivered it should take into account a multitude of factors that can inhibit or facilitate its implementation including training staff, information sessions for patients and caregivers and ensuring that all HCPs involved are collaborating, informed and supportive of the intervention. 

PPIE is an integral part of this study individuals with lived experience of kidney disease and associated renal replacement therapies have and continue to contribute additional expertise and give valuable, novel insights which is reflected by their involvement in this publication. The results of our forthcoming qualitative phenomenological study which explores the lived experience of renal cachexia [124] will also ensure that the voices of patients with ESKD and their carers are carefully considered in the future implementation of the MMIEAD intervention. Overall, this project is designed to have a substantial impact for patients, informal carers and HCPs and aligns with the UK’s ‘National Service Framework for Renal Services’ [125] ‘Standard one: A patient-centred service’ to improve quality of life for individuals with renal disease and their carers promoted by The European Kidney Health Alliance [126], The UK Kidney Association Kidney [127] and Kidney Care UK [128]. The project’s focus on renal cachexia which affect approximately 16% of those diagnosed with kidney failure, and their informal caregivers, creates the potential for an extensive impact in terms of the number of direct stakeholders benefitting from the proposed intervention. 

This research has several limitations. Firstly, because there is not enough information about the effectiveness of multimodal interventions in the renal population therefore, we were not able to provide high-quality scientific evidence for all links in the causal pathway, in particular the psychosocial intervention. Furthermore, given the limited information available, our systematic review [13] focused on multimodal interventions across chronic diseases not specifically renal disease. In addition, although the ToC approach has helped to define the components of this complex intervention and we have taken steps to consider all aspects of the context, the resulting ToC map may be a simplified version of a complex reality. Despite these limitations, we have taken the essential steps through designing the ToC map to consider the necessary rationales, assumptions, preconditions and causal pathways to ensure optimal implementation success. Irrespective of the possible limitations, all decisions were made in collaboration with the key stakeholders as documented through steps 1–6.

The results of this study provide the basis for the further design and evaluation of the MMIEAD intervention for those with renal cachexia. As de Silva et al. explain developing a ToC is an iterative process of reflection and adaptation as difficulties to implementation arise and new evidence emerges, the pathway may be amended and strengthened throughout all phases of implementation [37]. In the following phase, we will test and possibly further adapt the ToC map and the intervention components in terms of their acceptability and feasibility and subsequently, we will evaluate MMIEAD’s effectiveness in a cRCT as illustrated in Supplementary Document 2: The MMIEAD (Multimodal, Integrative, Exercise, Anti-inflammatory and Dietary counselling) study flow chart. If MMIEAD does not influence the outcomes as expected, this ToC map will additionally help us to ascertain whether the lack of effectiveness of the intervention is due to specific components of the intervention design, problems with the implementation or ineffectiveness of the intervention. We believe by using the ToC approach this has created a pathway for a more thorough evaluation considering that other trials of complex interventions often fail to detect or report the reasons why interventions succeed or are ineffective [129].

If the MMIEAD intervention is successful, it will be necessary to consider its implementation into ‘real-life practice’. As stated, certain preconditions will be essential including the assurance of clinical equipoise and the commitment of HCPs to inter/intra disciplinary working and similarly the commitment of the renal population in adherence to the intervention. For the sustainable, long-term implementation of the MMIEAD intervention, it is also necessary to consider that initially, significant financial support will be required. With the planned economic analysis for the forthcoming cRCT this will inevitably help to ascertain the potential cost savings to health services when those with renal cachexia receive the MMIEAD intervention. Therefore, the costs incurred may be counterbalanced by cost savings, for example relating to reduced inpatient admissions. If the future trial is successful, this addition to the existing evidence may also inform: clinical guidelines for cachexia management; and the allocation of necessary funding to further encourage support from HCPs and patients alike to ensure the intervention is sustained in ‘real-life practice’.

## 6. Conclusions

Within this study, we developed a theoretical framework for a multimodal, integrative, exercise, anti-inflammatory and dietary counselling (MMIEAD) intervention, which allowed us to create a ToC map. We explain how MMIEAD should be implemented, and which intervention components should be part of this multimodal intervention to achieve its desired long-term outcomes and impact. The ToC map provides the first comprehensive rationale of how this multimodal intervention for cachexia management is proposed to attain the desired outcomes and impact, something that has not been illustrated by renal research before. We will use this learning in the further design of the MMIEAD intervention and its evaluation to discover in greater depth why, how and in what context the multimodal intervention works best for patients with renal cachexia. 

## Figures and Tables

**Table 1:** ToC terminology.

Terminology	Definition—Adapted from De Silva et al. [33]
Impact	The real-world change we are trying to achieve for those with renal disease and their caregivers.
Ceiling of accountability	The point at which we stop accepting responsibility for achieving those outcomes solely through the proposed intervention.
Long-term outcomes	The outcome that the programme is able to achieve on its own. This can inspire the choice for particular primary and secondary outcomes in the evaluation of the intervention.
Intervention	The different components of the complex intervention. They represent certain “actions” that need to be undertaken to bring about a certain result, intermediate outcome or precondition. These are “those things that the programme must do to bring about the outcomes”.
Preconditions	A precondition or intermediate outcome is a necessary requirement, condition or element that needs to be realised for the long-term outcome/impact to be achieved. In the context of the multimodal intervention, these preconditions are the precursors or requirements for the programme being deemed successful.
Assumptions	An external condition beyond the control of the project that must or is assumed to exist for the outcome to be achieved.
Rationales	The facts or reasons (based on evidence or experience) behind the choice of the intervention activities and links in the causal pathway.

**Table 2:** Steps to develop the ToC Map.

Step	Aim	Methods	Output
1.	To obtain key stakeholder views on the potential development of a multimodal intervention for renal cachexia.	An initial renal online cachexia workshop was held to discuss the prospect of a multimodal intervention for renal cachexia. The workshop included a range of key multidisciplinary stakeholders including an international collaboration of consultants and academics specialising in nephrology, nutrition, exercise, psychology and PPIE representatives. Subsequently multiple exchanges were conducted via email and online to discuss the development of and the ToC underpinning the proposed multimodal intervention.	The stakeholders provided expert patient knowledge and experience that reinforced the acceptability of the proposed multimodal intervention for renal cachexia. Through this workshop, impact, preconditions, pathways through which change could potentially be achieved and necessary resources were discussed. Areas of particular focus were formulated into preconditions and added insight into hypothesised causal pathways. These included: Patient burden Care planning/multidisciplinary care Information sessions Providing an evidence base for the intervention The research team working closely with the clinical team Through the initial meeting, stakeholders subsequently agreed to work collaboratively with the core research team. Through this collaboration a first draft of primary and secondary outcomes were created, and the intervention indicators drafted.
2.	To obtain HCP perspectives on current practices in cachexia management	A mixed-methods study (online survey and two focus groups) was conducted with those who provide care for patients with ESKD and cachexia (published elsewhere) to determine factors which may influence awareness, understanding and treatment practices when managing renal cachexia [12].	Through the mixed methods study, we added to the preconditions, assumptions, the design of the intervention and the outcomes. This mixed method study confirmed that: A disease specific intervention is urgently required for those with renal cachexia A psychosocial component is required in the multimodal intervention Preconditions must include the training of relevant HCPs in cachexia Quality of life must be included as a key outcome
3.	To identify and assess the effectiveness of multimodal interventions for cachexia management	A systematic review (published elsewhere) of multimodal treatments intended to alleviate and/or stabilise cachexia and severe wasting was conducted [13]. The review included all relevant trials published between 2008–2019, systematically assessed the quality of these studies and the effectiveness of multimodal interventions for cachexia management.	The results of systematic review provided evidence to support the intervention components and proposed long-term outcomes. The review confirmed: Multimodal interventions are effective in the treatment of renal cachexia [13]. Significant improvements in weight gain, body composition, and physical activity as well as functionality [17,18,20,21,27,29] In cancer, research in multimodal interventions has progressed further than other chronic diseases, which provides evidence relating to the potential usefulness of this approach for cachexia management [13,23,49,50,51,52,53] The studies which showed promise in regard to their study design and results therein [50,51,54,55,56,57] were used to draft an outline of the proposed multimodal intervention and further refined the proposed long-term outcomes.
4.	To develop and refine the components of the proposed multimodal intervention for the ToC Map	In consultation with key stakeholders using the body of our research team’s cachexia research (cancer cachexia [39,40,41,42,43,44], cardiac cachexia [16,45,46,47,48] and renal cachexia [2,3,12,17] and wider relevant research we refined the specific components of the multimodal intervention based on the best evidence to date for a renal population undergoing haemodialysis.	The collaboration of key stakeholders used the best evidence to date to further develop a rationale for the intervention, hypothetical links in the causal pathway through which change may happen and long-term outcomes. Although a summary of the hypothetical links in the causal pathway for those with renal cachexia are stated below. The key stakeholders emphasised that it is necessary to remember that the hypothetical effectiveness of the proposed intervention is because it is integrative, therefore each component is proposed to work synergistically with the others. Exercise: resistance exercise linked to increased muscle mass, strength, reduced frailty, improved functioning and QoL, e.g., [58,59,60]. Dietary counselling: linked to increased appetite, weight, inflammation scores and nutritional knowledge and status, e.g., [61,62]. Targeting inflammation through Ω-3 PUFA: linked to cardioprotective improved inflammatory markers, lipid profile, heart rate and mortality, e.g., [63,64,65,66,67], Psychosocial support: linked to reducing fatigue experience, increased motivation for individuals and QoL, e.g., [68,69]. This evidence provided a basis for the rationale, intervention development and confirmation of the long-term outcomes.
5.	To create a draft ToC map based on integration of output from steps 1–4.	Using outputs from steps 1–4, comparison with existing ToC maps from other research projects, implementation science literature (in general and about multimodal interventions), the core research team drafted the ToC map.	Based on Steps 1–4, the core research team drafted the ToC map, which included:(i) impact, (ii) ceiling of accountability, (iii) long-term outcomes, (iv) preconditions, (v) interventions, (vi) assumptions, (vi) rationale (vii) indicators and (viii) resourcesThe ToC Map and associated documentation was then disseminated to the key stakeholders with a request for feedback given their various areas of expertise.
6.	To develop and amend the ToC Map in consultation with key stakeholders.	Further consultation and exchanges with key multidisciplinary stakeholders which included an international collaboration of consultants and academics specialising in nephrology, nutrition, exercise, psychology and PPIE representatives to construct the final ToC map and associated material.	The key stakeholders responded with in-depth responses which were incorporated into a final draft of a ToC map. Post amendments, the ToC Map and associated documentation was circulated to key stakeholders again to ensure all comments and critique had been suitably amended/responded to. In response the final ToC Map (Figure 1) and associated documentation was confirmed.

**Table 3:** Long-term outcomes and measures.

Primary Outcome	
**Outcome**	**Measure**
Increase in physical functioning	Short Physical Performance Battery (SPPB) [113]
**Secondary Outcomes**	
ii. Increase in lean muscle mass	Measures of Mid Upper Arm Muscle Circumference (MUAMC) [114], Bioelectrical Impedance Analysis (BIA) [115]
iii. Increase in strength	Handgrip strength (HGS) measured by a handheld dynamometer
iv. Weight gain	Body weight will be measured under identical conditions (post dialysis) and using the same weighing scale.
v. Reduced fatigue	Functional assessment of chronic illness therapy-fatigue (FACIT-F) [116]
vi. Increased appetite	The (FAACT) Functional Assessment of Anorexia/Cachexia Therapy [116]
vii. Reduction in inflammatory biomarkers.	Routine clinical bloods (conducted for haemodialysis) will be used for biochemical data collection—Haemoglobin (Hb), serum albumin and C-reactive protein (CRP)
viii. Improved quality of life (overall)	European Quality of Life-5 Dimensions five-level (EQ-5D-5L) [117]
ix. Reduced clinical frailty	Clinical Frailty Scale (CFS) [118] Fried frailty phenotype criteria (FC) [119]
x. Improved survival (all-cause)	Patient records

## Data Availability

Not applicable.

## Supplementary Materials

The following supporting information can be downloaded at: https://www.mdpi.com/article/10.3390/healthcare10122344/s1, All supplementary materials used are noted below; Supplementary Table S1: Checklist for reporting ToC in Public Health Interventions [112]; Supplementary Document 2: The MMIEAD (Multimodal, Integrative, Exercise, Anti-inflammatory and Dietary counselling) study flow chart [130].

## Abbreviations

BAME	Black, Asian, minority ethnics
COPD	Chronic Obstructive Pulmonary Disease
cRCT	Cluster Randomised Controlled Trial
CRP	C-reactive protein
CTU	Clinical Trials Unit
ESKD	End-stage kidney disease
Hb	Haemoglobin
HCP	Health Care Practitioner
IDPN	Intradialytic parenteral nutrition
MHD	Maintenance haemodialysis
MRC	Medical Research Council
MMIEAD	Multimodal, Integrative, Exercise, Anti-inflammatory and Dietary counselling intervention
NSAIDs	Non-steroidal anti-inflammatory drugs
NIKPA	Northern Ireland Kidney Patients Association
NIS	Nationwide Inpatient Sample
ONS	Oral nutritional supplement
PEW	Protein Energy Wasting
PPIE	Personal and Public Involvement and Engagement
Ω-3 PUFA	Polyunsaturated Fatty Acids
PIS	Participant Information Sheet
QoL	Quality of Life
RCT	Randomised Controlled Trial
SIP	Study information pack
SLT	Social learning theory
TIDieR	Template for Intervention Description and Replication
ToC	Theory of Change
QoL	Quality of Life
QUB	Queen’s University Belfast
UK	United Kingdom
