# Supplementary material for: Developing an Evidence and Theory Based Multimodal Integrative Intervention for the Management of Renal Cachexia: A Theory of Change
Source: Healthcare (Basel). 2022 Nov 22;10(12):2344. doi: 10.3390/healthcare10122344 (PMC9777598; doi:10.3390/healthcare10122344)
Supplement: Supplementary file 1 [file healthcare-10-02344-s001.zip › healthcare-1985692-supplementary.pdf]

Table S1. Checklist for reporting ToC in Public Health Interventions [112].

1. Is the ToC approach defined?
a. Is a definition of ToC given by the authors?
b. Do the authors explain their reasons for using a ToC approach?
2. Is the ToC development process described?
a. Are the methods used to develop the ToC, such as stakeholder meetings and interviews, document reviews, programme observation, existing conceptual frameworks or published research, described?
b. Where stakeholders are involved, is it clear how many stakeholders participated, what their role is in relation to the intervention, how they were consulted (e.g., number of interviews, focus groups, ToC workshops) and the extent to which the consultations were participatory?
c. Is the method used to compile the data into a ToC described? (including how disagreements between stakeholders were resolved)
d. Is the extent to which stakeholders were able to validate the resultant ToC and were owners of the final product described?
3. Is the resultant ToC (or a summary thereof) depicted in a diagrammatic form and does it include?
a. The long-term outcome or impact of the intervention
b. The anticipated short and medium term outcomes and the process of change
c. The intervention components which happen at different stages of the pathway
d. The context of the intervention
e. Assumptions about how change would occur
f. Additional ToC elements such as indicators, supporting research evidence, beneficiaries, actors in the context, sphere of influence and timelines where relevant.
4. Is the process of intervention development from the ToC described?
a. Are the methods of how interventions were refined from the ToC to something which can be implemented described? (For example, further stakeholder workshops, interviews, systematic literature reviews)
5. Is the way in which the ToC was used to develop and implement the evaluation described?
a. Are evaluation research questions generated from the ToC?
b. Is the role of ToC in the design, plan or conduct of the evaluation clear?

c. Does the paper describe the extent to which the key elements described in the ToC were measured in the evaluation (i.e., impact, short and medium term outcomes and the process of change, context, assumptions and the intervention)?
d. Does the paper describe whether and how process indicators were used to improve the quality of the intervention?
e. Is the role of the ToC in the analysis of the results of the evaluation clear?
f. Is the role of ToC in the interpretation of the results of the evaluation described? (including the breakdown of programme theory, unanticipated outcomes and causation including the strength and direction of causal relationships)

Supplementary document 2: The MMIEAD (Multimodal: Exercise, Anti-inflammatory and Dietary counselling) study flow chart

The core research team constructed a draft flow chart (below) of the MMIEAD (Multimodal: Exercise, Anti-inflammatory and Dietary counselling) intervention to work in conjunction with the ToC Map and further disentangle the key features of its effectiveness [32]. Using the synthesised evidence and ToC Map an implementation plan was designed to deliver a Cluster Randomised Controlled Trial (cRCT) including an internal pilot, process evaluation and economic evaluation in six NHS renal units. The trial has been designed to determine the clinical and cost effectiveness of the MMIEAD intervention among adult patients, receiving Haemodialysis at risk of cachexia.

The overarching research question is whether the MMIEAD intervention, compared to standard care, significantly reduces the risk of cachexia in patients with kidney failure receiving haemodialysis and whether such as intervention is cost-effective.

The aims of the trial were agreed as follows:

- To determine whether the MMIEAD intervention is effective at stabilising or reversing pathological loss of muscle mass for patients compared to standard care over 12 weeks, measured by physical functionality.
- To assess the effects of MMIEAD on patient mortality, experience, tolerability, resource used, and cost effectiveness.

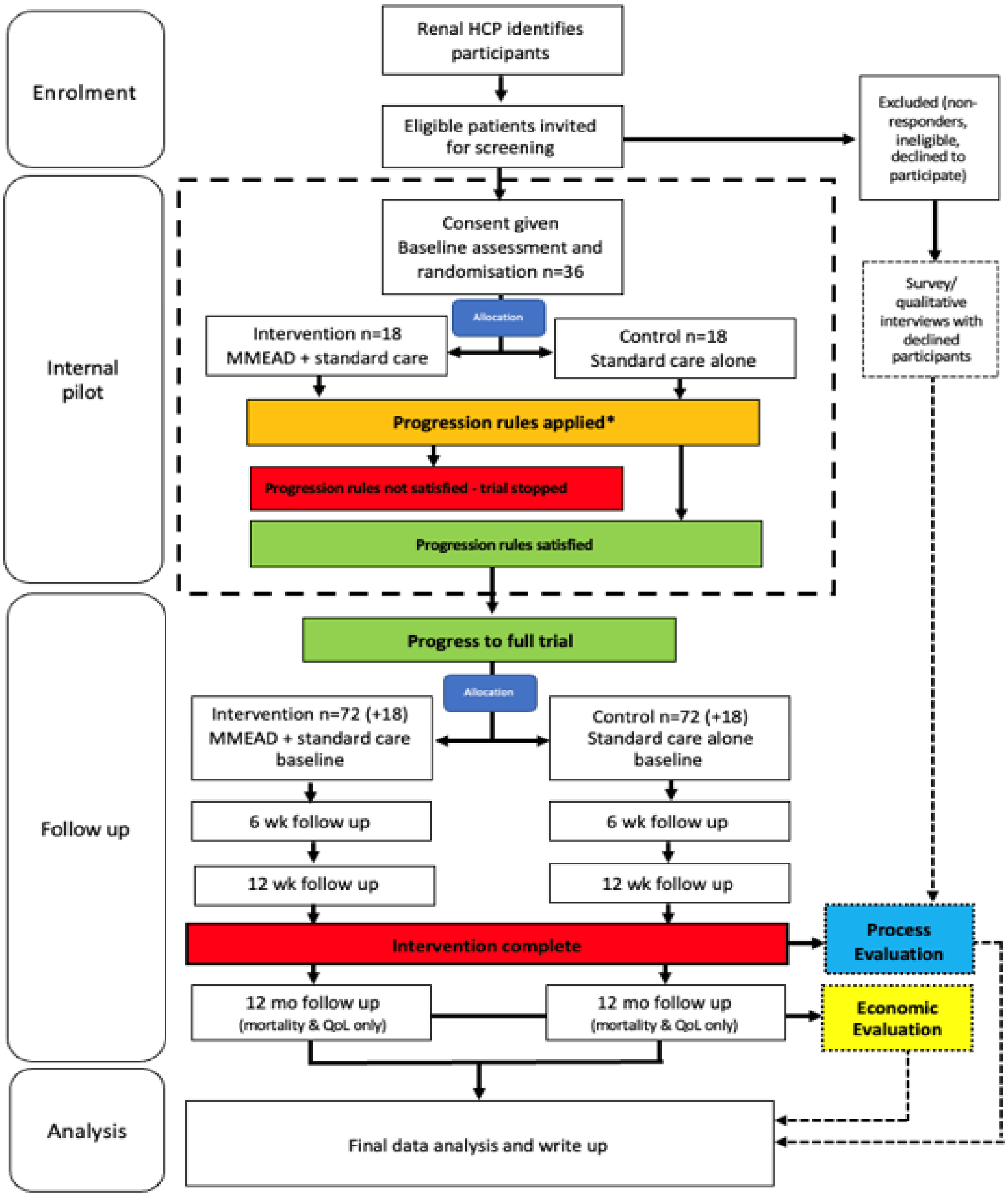

*Go: 75-100% recruitment: progress to main trial following a review of screening logs and protocol. Any barriers for recruitment will be addressed. Amend: 50-75% recruitment: progress to main trial with additional sites being recruited as well as a screening log and protocol review. Stop: less than 50% recruitment: the decision to progress will be made by the trial steering committee in association with HTA taking account of data completeness, consent rate, review of protocol deviations, violations, adverse events[24].

MMIEAD Study flow Chart

Process evaluation will be used to inform interpretation of outcome measures and will include assessments of implementation, mechanisms, and context [130]. The main trial will be preceded by a six-month internal pilot in two sites. We anticipate that by six months we will set up and recruit a minimum of 36 patients across the two sites. The pilot will be used to confirm recruitment adherence. This will ensure that any necessary adaptations to fit differing contexts, whilst allowing for flexibility, do not alter or undermine the active components and delivery of the intervention.
